# Supplementary material for: Differences in Demographics of Vaccinees, Access to, and Satisfaction with SARS-CoV-2 Vaccination Procedures between German General Practices and Mass Vaccination Centers
Source: Vaccines (Basel). 2022 Oct 28;10(11):1823. doi: 10.3390/vaccines10111823 (PMC9696883; doi:10.3390/vaccines10111823)
Supplement: Supplementary file 1 [file vaccines-10-01823-s001.zip › vaccines-1971671-supplementary.pdf]

vaccines

MDPI

Supplementary material

Figure S1a. Questionnaire translated into English: GP version.

Personal information				
1. Age	_____years			
2. Gender	□ Female	□ Male	□ Diverse	
3. What is your highest educational level?	□ University entrance qualification	□ Upper secondary education (grade 10)	□ Lower secondary education (grade 8 - 9)	
	□ Vocational qualification	□ No degree		
4. What is your highest professional qualification?	□ No professional qualification	□ Semi-skilled training		
	□ Apprenticeship	□ Vocational school		
	□ Specialized technical training	□ University degree		
5. Employment	□ Employed	□ Unemployed	□ Retired	
6. What is your current living situation? *(Please tick all that apply.)*	□ Living alone	□ Living with partner		
	□ Living with child(ren)	□ Shared accommodation		
7. Residence	□ City	□ Town	□ Rural area	
8. Do you have any pre-existing illnesses?	□ Yes	□ No		
If yes, please indicate:	1. ______________________________<br>2. ______________________________<br>3. ______________________________<br>4. ______________________________<br>5. ______________________________			
9. Do you get an influenza vaccination annually?	□ Yes, always	□ Yes, mostly	□ Yes, rarely	□ No, never
10. Have you already been infected with COVID-19?	□ Yes, in (month/year): ______/20_____	□ No		
11. Do you have a friend or family member who is/was infected with COVID-19?	□ Yes	□ No		
12. Do you know which prioritization group you belong to?	□ Yes	□ No	□ Not sure	
Which prioritization group do you think you belong to?	□ 1.	□ 2.	□ 3.	□ None

General questions about vaccination				
13. Which vaccination did you receive today?	□ First dose	□ Second dose		
Please indicate today's date: _____/______/2021 (day/month)				
If you received your second vaccine dose today, when did you receive the first dose? *(see vaccination certificate)*_____/______/20____ (day/month/year)				
14. Which vaccine type did you receive today? *(see vaccination certificate)*	□ BioNTech (Comirnaty®)	□ AstraZeneca (Vaxzevria®)	□ Moderna (Moderna®)	□ Johnson & Johnson (Janssen®)

If this was your second dose today, which vaccine type did you receive at your first vaccination?	□ BioNTech (Comirnaty®)	□ AstraZeneca (Vaxzevria®)	□ Moderna (Moderna®)	□ Johnson & Johnson (Janssen®)

15. How long did you wait for your **first** vaccination appointment at your GP practice (time between scheduling and vaccination date)?	□ < 1 week	□ 1-2 weeks	□ 3-4 weeks	□ 1-2 months	□ > 2 months

Access to vaccines			
16. Who took the first initiative to vaccinate?	□ The GP suggested the vaccination to me	□ I have approached my GP	□ Relatives/friends took the initiative
	□ Other:		
17. Had you already made an appointment at the vaccination center before today's vaccination at the GP practice?	□ **Yes**, I had already registered at the vaccination center and even made an appointment.		
	□ **Yes**, I had already registered at the vaccination center, but had **not yet** made an appointment.		
	□ **No**, I had not yet taken any steps to vaccinate at the vaccination center.		

18. How long did it take you to get to the GP practice?	□ < 5 min	□ 5-10 min	□ 11-20 min	□ 21-30 min	□ > 30 min
19. How did you come to the GP practice today?	□ Alone		□ Accompanied		
What mean of transport did you use to get to the GP practice today?	□ By foot	□ By bike	□ By car		
	□ By public transport				
	□ Other:				
20. Did you have any other concerns with your GP today?				□ Yes	□ No

Vaccination process				
21. How long did you wait in the GP practice today?	□ < 10 min	□ 10-30 min	□ 31-60 min	□ > 60 min
22. What were you informed about at the first and/or second vaccination? *(Please tick all that apply.)*	□ Vaccine type information		□ Vaccine benefits	
	□ Vaccine effectiveness		□ Behavior before/after vaccination	
	□ Common vaccination reactions, e.g., fever, shivering, heavy arm		□ Potential complications (exceeding normal level of vaccination reaction, e.g., allergic shock, sinus vein thrombosis)	
	□ Other:			
How much time was spent on patient education?	□ < 2 min	□ 2-5 min	□ 6-10 min	□ > 10 min
23. Was there an opportunity to ask questions after vaccination?	□ Yes	□ Yes, but I had none	□ Not sure	□ No

Attitudes and opinions					
**24. Please tick to what extent you agree with the statement.** **1= Strongly disagree 5= Strongly agree**					
	**1**	**2**	**3**	**4**	**5**

It was **important** for me to be vaccinated by my GP.	☐	☐	☐	☐	☐
It was **difficult** for me to get a vaccination appointment.	☐	☐	☐	☐	☐
The distance I had to travel to get vaccinated was **too far**.	☐	☐	☐	☐	☐
The access was **barrier-free**.	☐	☐	☐	☐	☐
The vaccination procedure was **easy** to understand.	☐	☐	☐	☐	☐
The doctor took **enough time** for the patient education.	☐	☐	☐	☐	☐
The doctors' information was **easy** to understand.	☐	☐	☐	☐	☐
I would **recommend** being vaccinated at my GP practice.	☐	☐	☐	☐	☐
I would also get vaccinated against COVID-19 at the **vaccination center**.	☐	☐	☐	☐	☐

**25. Please tick which statement applies to you.**										
	1	2	3	4	5	6	7	8	9	10
How stressful did you perceive the vaccination process?<br>**1= Very stressful │ 10= Not stressful at all**	☐	☐	☐	☐	☐	☐	☐	☐	☐	☐
How satisfied were you with the vaccination education?<br>**1= Not at all satisfied │ 10= Very satisfied**	☐	☐	☐	☐	☐	☐	☐	☐	☐	☐
How did you perceive your stay at the GP practice after the vaccination?<br>**1= Not at all pleasant │ 10= Very pleasant**	☐	☐	☐	☐	☐	☐	☐	☐	☐	☐

Figure S1b. Questionnaire translated into English: Vaccination center version.

Personal information				
1. Age	____years			
2. Gender	◻ Female	◻ Male	◻ Diverse	
3. What is your highest educational level?	◻ University entrance qualification	◻ Upper secondary education (grade 10)	◻ Lower secondary education (grade 8 - 9)	
	◻ Vocational qualification	◻ No degree		
4. What is your highest professional qualification?	◻ No professional qualification	◻ Semi-skilled training		
	◻ Apprenticeship	◻ Vocational school		
	◻ Specialized technical training	◻ University degree		
5. Employment	◻ Employed	◻ Unemployed	◻ Retired	
6. What is your current living situation? *(Please tick all that apply.)*	◻ Living alone	◻ Living with partner		
	◻ Living with child(ren)	◻ Shared accommodation		
7. Residence	◻ City	◻ Town	◻ Rural area	
8. Do you have any pre-existing illnesses?	◻ Yes	◻ No		
If yes, please indicate:	1. ____<br>2. ____<br>3. ____<br>4. ____<br>5. ____			
9. Do you get an influenza vaccination annually?	◻ Yes, always	◻ Yes, mostly	◻ Yes, rarely	◻ No, never
10. Have you already been infected with COVID-19?	◻ Yes, in (month/year): ______/20_____	◻ No		
11. Do you have a friend or family member who is/was infected with COVID-19?	◻ Yes	◻ No		
12. Do you know which prioritization group you belong to?	◻ Yes	◻ No	◻ Not sure	
Which prioritization group do you think you belong to?	◻ 1.	◻ 2.	◻ 3.	◻ None

General questions about vaccination				
13. Which vaccination did you receive today?	◻ First dose	◻ Second dose		
Please indicate today's date: _____/______/2021 (day/month)				
If you received your second vaccine dose today, when did you receive the first dose? *(see vaccination certificate)*_____/______/20____ (day/month/year)				
14. Which vaccine type did you receive today? *(see vaccination certificate)*	◻ BioNTech (Comirnaty®)	◻ AstraZeneca (Vaxzevria®)	◻ Moderna (Moderna®)	◻ Johnson & Johnson (Janssen®)

If this was your second dose today, which vaccine type did you receive at your first vaccination?	□ BioNTech (Comirnaty®)	□ AstraZeneca (Vaxzevria®)	□ Moderna (Moderna®)	□ Johnson & Johnson (Janssen®)

15. How long did you wait for your **first** vaccination appointment at the vaccination center (time between registration and vaccination date)?	□ < 1 week	□ 1-2 weeks	□ 3-4 weeks	□ 1-2 months	□ > 2 months

**Access to vaccines**						
16. How did the appointment process work after registering at the vaccination center?	□ I have made an appointment online			□ Friends/relatives have made an appointment		
	□ I have made an appointment by telephone			□ The vaccination center approached me		
	□ Other:					
17. How long did it take you to get to the vaccine center?	□ < 5 min	□ 5-10 min		□ 11-20 min	□ 21-30 min	□ > 30 min
18. How did you come to the vaccine center today?	□ Alone			□ Accompanied		
What mean of transport did you use to get to the vaccine center today?	□ By foot		□ By bike		□ By car	
	□ By public transport					
	□ Other:					

**Vaccination process**					
19. How long did you wait in the vaccine center today?	□ < 10 min	□ 10-30 min	□ 31-60 min	□ > 60 min	
22. What were you informed about at the first and/or second vaccination? *(Please tick all that apply.)*	□ Vaccine type information		□ Vaccine benefits		
	□ Vaccine effectiveness		□ Behavior before/after vaccination		
	□ Common vaccination reactions, e.g., fever, shivering, heavy arm		□ Potential complications (exceeding normal level of vaccination reaction, e.g., allergic shock, sinus vein thrombosis)		
	□ Other:				
How much time was spent on patient education?	□ < 2 min	□ 2-5 min	□ 6-10 min	□ > 10 min	
23. Was there an opportunity to ask questions after vaccination?	□ Yes	□ Yes, but I had none	□ Not sure	□ No	

Attitudes and opinions

24. Please tick to what extent you agree with the statement.
1= Strongly disagree 5= Strongly agree

	1	2	3	4	5
I would have **preferred** to be vaccinated by my GP.	□	□	□	□	□
It was **difficult** for me to get a vaccination appointment.	□	□	□	□	□

The distance I had to travel to get vaccinated was **too far**.	☐	☐	☐	☐	☐
The access was **barrier-free**.	☐	☐	☐	☐	☐
The vaccination procedure was **easy** to understand.	☐	☐	☐	☐	☐
The doctor took **enough time** for the patient education.	☐	☐	☐	☐	☐
The doctors' information was **easy** to understand.	☐	☐	☐	☐	☐
I would **recommend** being vaccinated at the vaccination center.	☐	☐	☐	☐	☐
I would go to the vaccination center **again** to be vaccinated against COVID-19.	☐	☐	☐	☐	☐

25. Please tick which statement applies to you.

	1	2	3	4	5	6	7	8	9	10
How stressful did you perceive the vaccination process?<br>**1= Very stressful │ 10= Not stressful at all**	☐	☐	☐	☐	☐	☐	☐	☐	☐	☐
How satisfied were you with the vaccination education?<br>**1= Not at all satisfied │ 10= Very satisfied**	☐	☐	☐	☐	☐	☐	☐	☐	☐	☐
How did you perceive your stay at the vaccine center after the vaccination?<br>**1= Not at all pleasant │ 10= Very pleasant**	☐	☐	☐	☐	☐	☐	☐	☐	☐	☐

Figure S2. Participants' preference for their chosen vaccine site and their desired vaccine site. Values range from 1 = strongly disagree to 5 = strongly agree.

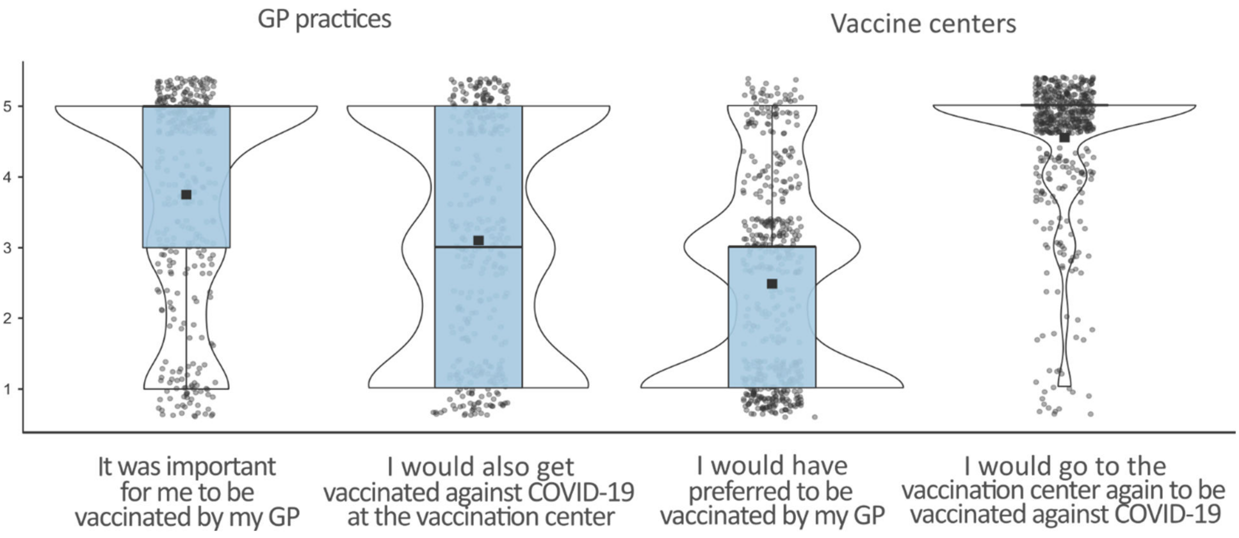

Text S3. Results of the assumption checks for multiple linear regression models.

<u>Model 1, satisfaction with patient education:</u>

Multicollinearity was not a concern in our model (highest correlation between dependent and independent variables was r = .55 and VIF ranged between 1.03 and 2.99). The data met the assumption of independent residuals (Durbin-Watson = 1.935). The histogram and p-p plot of standardized residuals indicated that the data contained approximately normally distributed residuals with minimal deviation from the line. The scatterplot of standardized residuals showed that the data met the assumptions of homogeneity of variance and linearity. All participants in the model showed Cook's Distance values below 1, wherefore all were included.

<u>Model 2, recommendation of vaccination at patients' vaccination site:</u>

Multicollinearity was not a concern in our model (highest correlation between dependent and independent variables was r = .62 and VIF ranged between 1.08 and 3.36). The data met the assumption of independent residuals (Durbin-Watson = 1.872). The histogram and p-p plot of standardized residuals indicated that the data contained approximately normally distributed residuals with minimal deviation from the line. The scatterplot of standardized residuals showed that the data met the assumptions of homogeneity of variance and linearity. All participants in the model showed Cook's Distance values below 1, wherefore all were included.
